# Monocrotaline toxicity in rats: decreased lung retinol and elevated alpha-tocopherol levels in lung and liver

**DOI:** 10.1017/jns.2026.10077

**Published:** 2026-02-06

**Authors:** Richard Carleton Baybutt, Vance La Baron Smith, Donovan Gabriel Kearns, Samuel Bryce Stafford

**Affiliations:** 1 Department of Human Nutrition, Kansas State University, Manhattan, KS, USA; 2 Department of Applied Health Science, Wheaton College, Wheaton, IL, USA; 3 Department of Nutrition Science, East Carolina University, Greenville, NC, USA

**Keywords:** Alpha-tocopherol, Liver, Lung, Monocrotaline, Vitamin A

## Abstract

Monocrotaline (MCT) induces lung injury and pulmonary hypertension (PH) by a mechanism that is in part due to oxidative stress. The purpose of this study was to determine how MCT affected nutrient antioxidants retinol and alpha-tocopherol in a rat lung and liver. Rats were fed a purified diet (AIN-93G) one-week prior to a subcutaneous injection of MCT (60 mg/kg) and remained on the diet throughout the study. Three weeks after injection, the animals were euthanized, and the lungs and livers were analyzed for retinol, alpha-tocopherol, phospholipid (PL), and cholesterol content. Lung retinol concentrations were significantly lower in MCT-treated rats, 2.0 ± 1.2 (nmol/g lung) vs. vehicle control (VEH), 5.8 ± 1.4 (*P* < 0.01). However, liver retinol concentrations were not significantly different, 3.3 ± 1.3 vs. 2.5 ± 0.9 nmol/g liver. Alpha-tocopherol was significantly greater in MCT-treated rats in the lung, 145 ± 24 vs. 99 ± 13 nmol/g lung (*P* < 0.001), and liver, 107 ± 30 vs. 47.7 ± 4.8 nmol/g liver (*P* < 0.001). Phospholipid and cholesterol were significantly lower in the lung of the MCT-treated group, but not significantly different in the liver. In conclusion, retinol along with phospholipid, and cholesterol were decreased in the lungs whereas alpha-tocopherol was elevated in the lungs and liver in response to MCT. These findings along with others suggest a novel mechanistic link between MCT-induced oxidative stress, lung vitamin A depletion, inflammation and the impairment of alveolar cell proliferation and repair. Pulmonary retinol is important in the pathogenesis of MCT-induced lung injury.

## Introduction

Monocrotaline (MCT) is a pyrrolizidine alkaloid found in the plant seeds of the species *Crotalaria spectabilis* ^(1)^ and has been used to induce lung injury and pulmonary hypertension (PH) in rats. Cytochrome P450 (CYP) enzymes, specifically the CYP3A subfamily, are responsible for the hepatic bioactivation of monocrotaline (MCT) into its reactive metabolite, dehydromonocrotaline (MCTP), which is the major pathway for activation^(2–4)^. MCTP has been found to cause oxidative stress in both hepatic and pulmonary tissues^(5–7)^. The oxidative metabolite MCTP is transported from the liver to the lungs, increasing lung oxidation as evidenced by marked increases of oxidative stress markers in lung tissues^(2,3)^. The increased oxidative stress markers induced by MCT include elevated lipid peroxidation byproducts: malondialdehyde (MDA), 4-hydroxynonenal (HNE-4) and 8-isoprostane^(5,8–12)^. Also, reactive oxygen species (ROS) byproduct nucleic acid 8-hydroxy-2’-deoxyguanosine (8-OHdG) was found to be elevated^(9)^. All of the subsequent antioxidant enzyme activity are downregulated, which is indicated by lower levels of superoxide dismutase (SOD), catalase, total sulfhydryl groups (SH), glutathione peroxidase, and glutathione (GSH)^(10,12–15)^. The oxidative stress exhibited in the lungs is linked with PH and then ultimately right ventricular hypertrophy (RVH)^(2)^.

MCT-induced PH has provided scientists with an excellent animal model for the study of PH since 1967, which has given insights on PH progression and has been used for preclinical testing of new therapeutic strategies^(1–3,8–10,16–18)^. MCTP causes severe vascular remodeling after initial onset of injury^(2)^. Pulmonary vascular endothelial cell injury is accompanied by progressive pulmonary vascular leakage as well as the inhibition of endothelial cell division^(3)^. Extensive endothelial cell injury results in the migration of inflammatory cells, such as neutrophils, to the area. Polymorphonuclear leukocytes release free radical components, which can damage alveolar cell membrane components, resulting in cell death^(16)^. An effective means of combating MCT-induced oxidative stress involves the antioxidant system. Retinol is a well-known antioxidant^(19)^. However, the precise role of vitamin A in the pathology associated with monocrotaline is not thoroughly defined.

Vitamin A and its metabolites are necessary cofactors that regulate gene expression and cellular differentiation^(3)^. Within the liver, vitamin A mainly functions as a stored nutrient, although it has been shown to provide an anti-inflammatory role as well^(20)^. Vitamin A has also been shown to influence male albino rat sensitivity to hexacholorocyclohexane (HCH) toxicity by modulating the activity of hepatic xenobiotic metabolizing enzymes, thus vitamin A may function to assist in the detoxification of hepatotoxins^(21)^. The pleotropic effects of vitamin A are well known, however vitamin A’s role as an anti-inflammatory and detoxifying agent in response to MCT is less well known.

There is evidence to suggest that oxidative injury is increased in liver disease. Isoprostanes, a family of prostaglandin isomers, are formed in a free radical catalyzed manner from arachidonic acid-containing phospholipid^(22)^. Data from this study showed that isoprostane biosynthesis, and subsequent oxidative injury, increased in patients with hepatic cirrhosis^(22)^. Diseases involving oxidative stress and inflammation, such as tumorigenesis and cancer development, have been shown to alter alpha-tocopherol content in tissue where damage occurs. Previous studies have shown that alpha-tocopherol content was depleted in the presence of hepatocellular carcinoma, but alpha-tocopherol concentration doubled in metastatic liver nodules in patients with liver cirrhosis^(23)^. The generation of free radicals due to hypoxic gas mixture resulted in depletion of glutathione and alpha-tocopherol concentrations in the liver^(24)^. As an antioxidant, alpha-tocopherol protects the unsaturated fats from oxidation in low density lipoproteins (LDLs)^(25)^.

The aim of this research was to determine how MCT toxicity alters lung and liver content of retinol and alpha-tocopherol content in rats. The effects of pyrrolizidine toxins on vitamin A and alpha-tocopherol concentrations are not known. MCT toxicity presents a model for systemic toxicity that can be used to determine how antioxidant and anti-inflammatory compounds are utilized, in particular retinol and alpha-tocopherol.

## Materials and methods

### Animals and treatment

Male Sprague-Dawley rats (Harlan Sprague Dawley, Indianapolis, IN) were housed in stainless steel cages at approximately 24°C with a 12-hour light: 12-hour dark cycle. Animal care and use were approved by the Institutional Animal Care and Use Committee of Kansas State University. Rats were cared for in an animal facility approved by the American Association for the Advancement of Laboratory Animal Care.

Monocrotaline (Trans World Chemicals, Rockville, MD) was dissolved in 0.1 mol/L HCl and neutralized with 0.1 mol/L NaOH. Rats were fed their respective diets for 1 week and then injected subcutaneously with 60 mg monocrotaline/kg body weight or its vehicle (VEH).

Twelve rats weighing 150–170 g were distributed randomly into two groups, MCT and VEH, and were injected with monocrotaline or its vehicle. The sample size was chosen based on previous experiments. All rats were pair-fed the control AIN-93G growth purified diet (Dyets, Bethlehem, PA). AIN-93G includes 2.2 mg of vitamin A (as retinyl palmitate) and 75 mg of vitamin E (as alpha-tocopheryl acetate) per kilogram of diet. Food intake and body weights were measured.

Twenty-three days after injection of MCT or its VEH, both groups were anesthetized with halothane (U.S.P., Halocarbon Laboratories, River Edge, NJ), and heart, lung, and liver were collected and stored in a −70°C freezer until analysis. Prior to analysis, butylated-hydroxytoluene, BHT, (2,[6]-Di-tert-butyl-p-cresol, Sigma Chemical Co, St. Louis, MO) was added, and tissues were minced. Three aliquots of 500 mg of minced tissue were stored in amber-colored centrifuge tubes on ice and then analyzed.

### Heart measurements

The hearts from each rat were analyzed to determine the severity of right ventricular hypertrophy (RVH). The right ventricle (RV) was separated from the left ventricle (LV) plus septal wall (S), and both parts were weighed to assess RVH.

### Total lipid fraction

Total lipid fractions were separated by the Folch method^(26)^. Isolated lipid extracts were sealed and stored in a dark −70°C freezer until lipid analysis.

### Cholesterol determination

Three hundred microliters of 33% KOH (Fisher Scientific, Fair Lawn, NJ) in deionized water, 100 µL of total lipid extract and 3 mL 95% ethanol (HPLC grade, Fisher Scientific) were pipetted into tubes that were then capped and vortexed. Lipid extracts were saponified for 15 min in a 60°C water bath, removed, and allowed to cool. Five mL of hexane (HPLC grade, Fisher Scientific) and 1.5 mL of deionized water were added to the tubes, and the upper lipid extract layer was pipetted off and placed in a second set of tubes. The solvent was evaporated with nitrogen gas (Compressed, Salina, KS). Three mL of 50 mg/100 mL ortho-phthalaldehyde reagent (Sigma Chemical Co.) in glacial acetic acid (Fisher Scientific) was added to the tubes and vortexed immediately. Tubes were read by spectrophotometry (UV-1201, UV-VIS Spectrophotometer, Shimadzeu Corp), at an optical density of 550 nm. Absorbance was measured against the standard curve of cholesteryl palmitate (Sigma Chemical Co., in chloroform) at a concentration of 1 mg/mL as a reference standard.

### Phospholipid determination

One hundred microliters of total sample was added to each test tube and evaporated with nitrogen gas. Four hundred microliters of chloroform (HPLC grade, Fisher Scientific), 100 µL of chromogenic solution was added to lipid extract and gently vortexed^(27)^. Tubes were tightly capped and placed in a boiling water bath for 1–1.5 min. Samples were removed from the water bath, and after reaching room temperature, 4 mL of chloroform was added, tubes were vortexed, then allowed to sit for 30 min. The lower solution was recovered by pipetting, and the amount of phospholipid was measured spectrophotometrically at 710 nm. The concentration of phospholipid was determined by measuring absorbance against a standard curve of phosphatidylcholine (Sigma Chemical Co.).

### Measurement of alpha-tocopherol concentration

Homogenizer tubes were pre-weighted, and approximately 100–200 mg of minced tissue sample was placed in the tube. Six hundred micromoles of internal standard and alpha-tocopherol acetate (Sigma Chemical Co.) dissolved in methanol (HPLC grad, Fisher Scientific) were added to the tubes. Two mL of acetone (HPLC grade, Fisher Scientific) was added, and the contents of the tube were homogenized. This step was repeated twice to ensure that the contents were thoroughly homogenized. Test tubes were centrifuged for 20 min, and using a 13 mm syringe and syringe filter (0.45 µm PTFE, Alltech) the supernatant was pipetted from the original tubes and filtered into another set. The original tubes were washed with acetone twice, and their contents were nitrogen evaporated. Six hundred microliters of methanol (Fisher Scientific) was added to the tubes to re-dissolve the alpha-tocopherol. Fifteen microliters of the methanol/alpha-tocopherol solution was injected into the high-performance liquid chromatography (HPLC) column with a retention time of 7 min.

### Measurement of retinol concentration

Two hundred microliters of total sample and 10 mL of KOH solution (95.0% ethanol, HPLC grade, Fisher Scientific, 5.0% KOH, pelleted, Fisher Scientific, 1.0% pyrogallol, Sigma Chemical Co.) were added to each test tube and vortexed. The mixture was saponified for 20 min in a 60°C water bath and cooled to room temperature. Twenty mL of hexane and 10 mL of water were added and the tubes were vortexed again. The upper hexane layer was pipetted off and 100 µL of retinyl acetate (all trans-retinol acetate, Sigma Chemical Co.) was added as an internal standard. The solution was evaporated with nitrogen and brought to a final volume of 100 µL with methanol (HPLC grade, Fisher Scientific). Fifteen microliters of retinol extract were injected into HPLC with a retention time of 7 min.

### The use of HPLC analysis of alpha-tocopherol and retinol

High performance liquid chromatography (HPLC) was used to analyze alpha-tocopherol and retinol. HPLC analysis used two necessary phases, the mobile and stationary phases. The mobile phase was a mixture of substances to be fractionated, which in the case of our research contained the sample and an internal standard, either retinyl acetate or alpha-tocopherol acetate. The stationary phase consisted of a porous solid matrix, in our case a C-18 column, which the aforementioned mobile phase was passed over. The HPLC was set for reverse phase chromatography, in which the stationary phase was less polar than the mobile phase. This resulted in a lower affinity of polar molecules in the mobile phase than the stationary phase. The HPLC was set to ensure a higher percent recovery (elution) of the internal standard, as well as the micronutrients under investigation, alpha-tocopherol and retinol.

### Statistical analysis

When appropriate, data were expressed as means ± standard error of the mean (SEM). Statistical differences among means were considered significant when *P* < 0.05. Treatment-dependent changes for all reported values, including organ weights and the right ventricular hypertrophy data [RV/(LV + S)], were analyzed using T-tests to determine differences between the control (VEH) and monocrotaline (MCT) group means.

## Results

**Figure 1.**
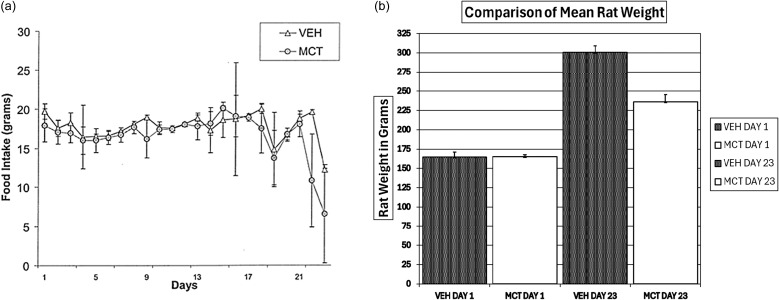
Food consumption and rat weight. (a) Food consumption for pair-fed animals, VEH and MCT groups after injection of MCT or its vehicle. Animals were fed a standard AIN 93G diet. The pair feeding was done as follows: the average food intake for the MCT group was provided to the VEH group the following day. Data shown ranges from day 1 through day 23. All values are expressed as mean ± SEM. n = 6. (b) On day 1 of the study the animals in both groups were very similar in weight, VEH weighing 164.7 ± 6.3 and MCT weighing 165.3 ± 2.5 g. By day 23, the average weights of both groups were statistically significantly different, VEH weighing 300.5 ± 8.4 and MCT weight 236.2 ± 9.5 g (*P* < 0.00001).

Food intake between both groups was consistent and not significantly different for the first 21 days after injection of MCT or its vehicle, because both groups were pair-fed (Figure 1a). Two MCT-treated rats died on day 21 and a parallel experiment for the two MCT-treated replacement rats was carried out using the same breed, age, food and length of time after MCT injection before tissue collection. The results from the two replacement MCT-treated rats’ analysis showed that their food intakes were not significantly different from those of the 4 surviving MCT-treated rats, therefore their data was combined with the original MCT-treated rats.

On day 22 there was an abrupt change in food consumption; the MCT group consumed approximately half the food intake as the control group, VEH, 19.6 ± 0.3, vs. MCT, 10.8 ± 5.9 g. Decreased food consumption was accompanied by labored breathing and lengthened response time to external stimuli. Weight gain was also significantly affected by monocrotaline toxicity (Figure 1b). At the start of the study animals in both groups were very similar in weight, VEH weighing 164.7 ± 6.3 and MCT weighing 165.3 ± 2.5 g. By day 23, the average weights of both groups were significantly different, VEH weighing 300.5 ± 8.4 and MCT weight 236.2 ± 9.5 g (*P* < 0.00001).

**Figure 2.**
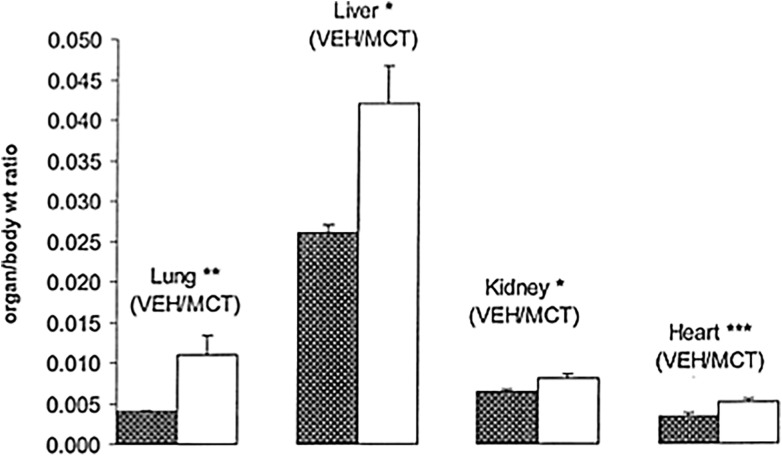
Comparison of various organs expressed as organ to body weight ratio 3 weeks after MCT/VEH injection. Values expressed as mean ± SEM, n = 6. Statistically significant differences in organ/body ratio are shown: *P < 0.0005, **P < 0.0001, ***P < 0.00005.

There was no significant difference in organ weights except for lungs (*P* < 0.001) and hearts (*P* < 0.002). Because MCT-treated rats weighed significantly less than the VEH group, we expressed the organ weights relative to body weight. The organ to body weight ratio for the harvested organs was significantly heavier for the MCT group compared to VEH (Figure 2). The lung ratio for VEH was 0.0042 ± 0.0002 and for MCT was 0.0108 ± 0.0023 g (*P* < 0.00004), and the liver ratio for VEH was 0.026 ± 0.001 while for MCT it was 0.037 ± 0.0047 g (*P* < 0.0002). The kidney ratio for VEH was 0.0064 ± 0.0003 and for MCT it was 0.0077 ± 0.005 g (*P* < 0.0004). The heart VEH ratio was 0.0034 ± 0.0003 and MCT was 0.0051 ± 0.0004 g (*P* < 0.00001).

**Figure 3.**
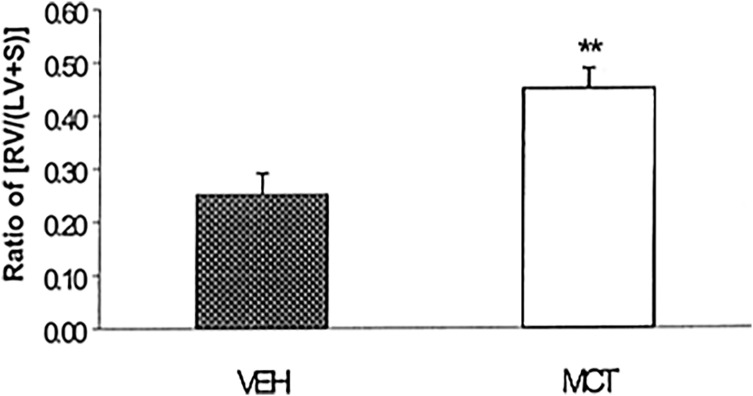
Development of right ventricular hypertrophy in rats 3 weeks after subcutaneous injection of MCT or its Vehicle. Severity of disease assessed by RVH: (RV/[S + LV]). Values expressed as mean ± SEM, n = 6. MCT treatment group showed RVH greater than 0.3, **P < 0.001. Note: This cardiac ratio has no units. RV: Right ventricle; S: Septum; LV: Left Ventricle.

RVH was determined as an indirect measure of PH (Figure 3). RVH for VEH was 0.25 ± 0.041, and MCT was calculated to be 0.45 ± 0.038 (*P* < 0.001). A ratio of approximately 0.2 was considered normal and a ratio of 0.3 as hypertensive^(28)^.

**Figure 4.**
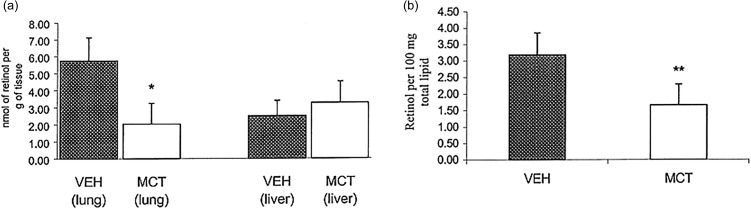
Retinol. (a) Concentration of retinol per gram of tissue from MCT and VEH rats. Values expressed as mean ± SEM, n = 6. Lung concentrations of retinol were significantly different, *P < 0.001. Liver concentrations were not statistically significant between groups. (b) Retinol, expressed per 100 mg total lipid in MCT and VEH rats. Retinol content is significantly lower in the lungs of MCT treated rats, **P < 0.003. All values expressed as mean ± SEM, n = 5 or 6.

Retinol concentrations in the lungs of MCT treated rats were significantly lower than VEH (Figure 4a), VEH lung concentration was 5.8 ± 1.4 vs. MCT, 2.0 ± 1.2 nmol/g lung tissue (*P* < 0.001). Liver concentrations did not differ significantly, VEH at 2.5 ± 0.9 vs. MCT at 3.3 ± nmol/g liver tissue. As retinol is a fat-soluble vitamin, we expressed it per 100 mg total lipid (Figure 4b). The data shows that there were significantly lower levels of retinol per lipid in the lungs of MCT-treated rats, MCT was 1.66 ± 0.63 and VEH 3.18 ± 0.67 nmol retinol/100 mg total lipid (*P* < 0.001).

**Figure 5.**
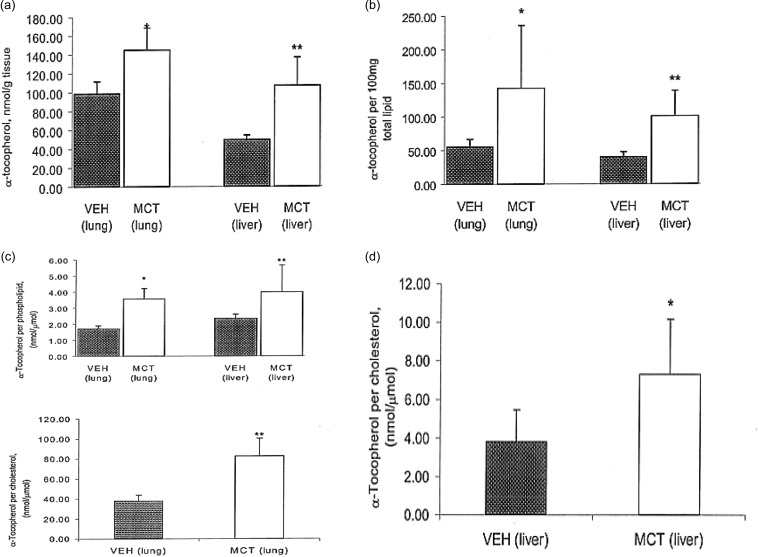
Alpha-tocopherol. (a) Alpha-tocopherol expressed nmol per gram of tissue. Alpha-tocopherol is significantly higher in both lung and liver of MCT treated rats, compared to VEH rats, *P < 0.01, **P < 0.005. All values expressed as mean ± SEM, n = 5 or 6. (b) Alpha-tocopherol expressed per 100 mg total lipid. Alpha-tocopherol/total lipid ratio is significantly higher in the lung and liver of MCT-treated rats compared to VEH, *P < 0.05, **P < 0.01. All values expressed as mean ± SEM, n = 5 or 6. (c) Alpha-tocopherol expressed nmol per µmol of phospholipid and cholesterol in MCT and VEH treated rats. Ratios of alpha-tocopherol to phospholipid and cholesterol are both significantly higher in the lung and liver of MCT-treated rats compared to VEH, *P < 0.01, ** *P* < 0.05. All values expressed as mean ± SEM, n = 5 or 6. (d) Alpha-tocopherol expressed as nmol/µmol of cholesterol in the liver of MCT and VEH treated rates. Alpha-tocopherol/cholesterol ratio is significantly higher in MCT treated rats, as compared to VEH, *P < 0.05. All values expressed as mean ± SEM, n = 5 or 6.

Although there was a decrease in retinol concentration in the lungs, there was a surprising increase in lung and liver concentrations of alpha-tocopherol (Figure 5a). Lung concentration of alpha-tocopherol in MCT treated rats (145 ± 24 nmol g/tissue) was approximately 30% higher than VEH, 99 ± 13 nmol g/tissue (*P* < 0.01). Liver content of alpha-tocopherol for MCT was 107 ± 30 nmol g/tissue vs. VEH was 47.7 ± 4.8 nmol g/tissue. Alpha-tocopherol, expressed per total lipid (Figure 5b), was found to be 65% higher in the lungs of MCT treated rats, 143 ± 93 vs. VEH at 55 ± 11 nmol vitamin E/100 mg total lipid (*P* < 0.05). In the liver MCT, 101 ± 37.7 vs. VEH, 40 ± 7.6 nmol vitamin E/100 mg total lipid (*P* < 0.01).

Alpha-tocopherol, expressed in nmol per µmol of phospholipid, was higher in MCT treated rats in the lung, with MCT was 3.6 ± 0.7 vs. VEH was 1.7 ± 0.2, as well as in the liver, with MCT value was 4 ± 1.7 vs. VEH was 2.34 ± 0.3 (*P* < 0.05). Alpha-tocopherol, expressed as nmol per µmol cholesterol, was also higher in the lung of MCT treated rats, with MCT was 82.7 ± 18 vs. VEH was 38 ± 56 (*P* < 0.0001) (Figure 5c). In the liver the MCT value was 7.3 ± 2.9 vs. VEH was 3.8 ± 1.6 (*P* < 0.05) (Figure 5d).

**Figure 6.**
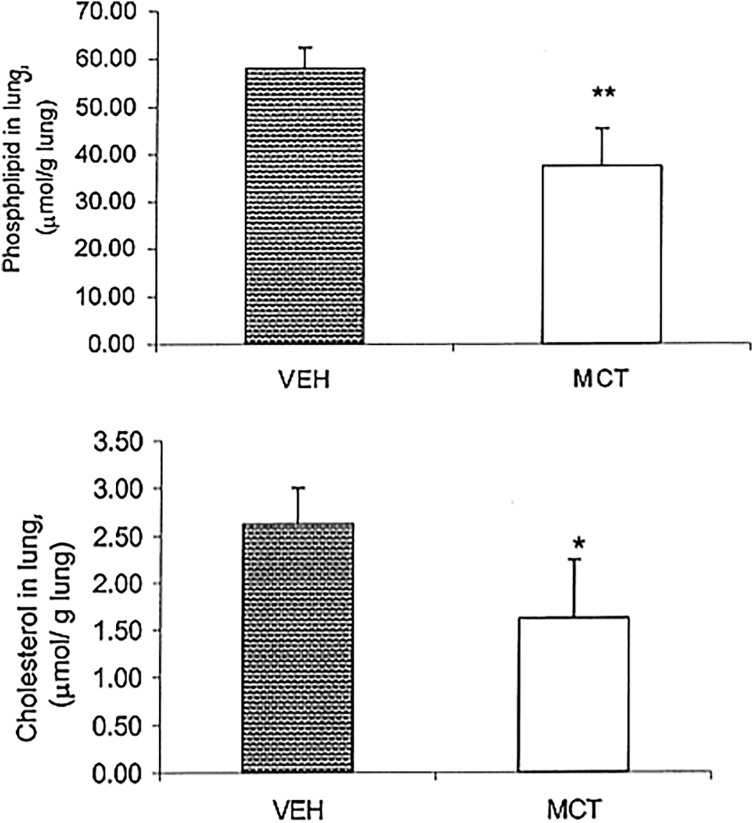
Phospholipid and cholesterol in the lung, (µmol/g lung) of MCT and VEH treated rats. Phospholipid and cholesterol were both significantly lower in MCT treated rats, **P < 0.0005, *P < 0.007. All values expressed as mean ± SEM, n = 5 or 6.

Although cell membrane concentrations of phospholipid and cholesterol were not measured directly, total lung and liver concentrations of these two important fats were determined (Figure 6). Despite higher concentrations of alpha-tocopherol in the lung and liver of MCT treated rats, total phospholipid and cholesterol in the lung (Figure 6) were significantly lower in MCT compared to VEH. Lung PL for MCT was 37.5 ± 8.6 and was 58 ± 4.4 µmol/g lung tissue for VEH. Cholesterol concentrations were also significantly lower in MCT rats, 1.6 ± 0.6 vs. VEH was 2.6 ± 0.4 µmol/g lung tissue (*P* < 0.01). Liver concentrations of phospholipid and cholesterol were not found to be significantly different.

## Discussion

We investigated how the oxidative toxin, monocrotaline that is used to induce pulmonary hypertension in a rat model, changes retinol and alpha-tocopherol concentrations within the lung and liver of rats. Retinol plays an important role in the normal lung function. We also measured cholesterol and phospholipid concentrations in the lungs and liver of rats, due to their significance in the lung surfactant producing cell, the type II pneumocyte. The results of this study for the first time showed that MCT; 1) decreased retinol content in the lung but did not change liver retinol content; 2) increased alpha-tocopherol content in both the lung and liver; and 3) decreased phospholipid and cholesterol content of the lungs but did not significantly alter those lipid compounds in the liver.

This is the first time reported that vitamin A levels within the lungs were decreased in response to MCT toxicity. Decreasing concentrations of lung retinol may be in response to oxidative stress in the lung^(19,29)^. We have previously shown that in a pro-oxidative and pro-inflammatory smoking model, vitamin A levels were depleted in lung and liver^(29)^. Further, several studies have shown that retinol concentration decreases the presence of lipid peroxidation that was induced by tissue inflammation^(30,31)^. Vitamin A depletion induced by MCT could be mediated in part through oxidation and/or inflammation.

In MCT-treated rats there is increased lung inflammation^(12,32)^. We have previously shown that vitamin A deficiency increases lung inflammation^(33)^. Furthermore, in another study we have shown that in MCT-treated rats that are supplemented with additional vitamin A, inflammation is significantly reduced^(32)^. In this current study, we have shown that MCT significantly reduces vitamin A in the lung. Therefore, the reason that there is an increase in lung inflammation in MCT-treated rats may in part be attributed to depleted vitamin A. It is important to note that vitamin A supplementation did not prevent right ventricular hypertrophy in our pulmonary hypertension model, but effective decreased lung inflammation^(32)^.

As we have previously noted, MCT increases inflammation and oxidation, depleting retinol. The increased inflammation may also decrease retinol transport due to the adverse effect on the transport protein for vitamin A, retinol binding protein (RBP). Monocrotaline has been shown to cause acute inflammation in liver tissues^(34)^. Other studies have shown that acute systemic inflammation significantly reduces serum RBP and retinol, and both hepatic RBP’s mRNA and protein concentrations^(35,36)^. Likewise, it has been shown that there is an inverse relationship between plasma inflammatory markers such as CRP and serum RBP^(37)^. The decrease in RBP was paralleled with a decrease in plasma retinol levels^(36,37)^. Retinol binding protein is the major transport protein for retinol from the liver, and it is responsible for the transport of retinol throughout the blood^(35–37)^. Decreased concentrations of RBP will decrease the release of hepatic retinol, potentially elevating hepatic retinol, which has been previously reported^(36)^. However, for our pro-oxidative induced MCT model, we observe no change in hepatic retinol, which may be explained by oxidative destruction of the retinol. This warrants future research.

This is the first time it has been reported that concentrations of alpha-tocopherol were elevated in the liver and lung in rats treated with MCT. Elevated lung and liver alpha-tocopherol levels have been reported in other pro-oxidation models^(38–40)^. It is well known that alpha-tocopherol is utilized by the body for its antioxidant capabilities and studies have shown a positive correlation between oxidative stress within the lung and elevation of alpha-tocopherol^(38,39,41,42)^. Oxidative stress appears to be one of the key mechanisms by which MCT exerts its toxic effects^(2,3,5–12,15)^. With regard to MCT toxicity, it seems that the increase in tissue alpha-tocopherol is in response to oxidative metabolites.

Total lung phospholipid and cholesterol were found to be significantly lower within the MCT treated group (Figure 6). Eighty-six percent of lung phospholipid is made up of cellular phospholipid^(43)^. Type II pneumocytes are the most numerous alveolar cells and are markedly decrease in response to MCT administration^(44, 45)^. Both phospholipid and cholesterol are critical components of the type II pneumocyte^(45,46)^. Previously, it has been shown that retinoic acid promotes cell proliferation of type II pneumocytes in the cell repair process^(47,48)^. MCT-induced decrease in the lung cell number would also decrease total lung phospholipid and cholesterol, which may be attributed to depleted Vitamin A.

## Conclusion

In summary, this study provides the first direct evidence that MCT-induced pulmonary toxicity is characterized by a significant and organ-specific dysregulation of key lipophilic micronutrients, retinol and alpha-tocopherol. We demonstrate that MCT administration selectively depletes pulmonary retinol while concurrently elevating alpha-tocopherol concentrations in both the lung and liver.

**Figure 7.**
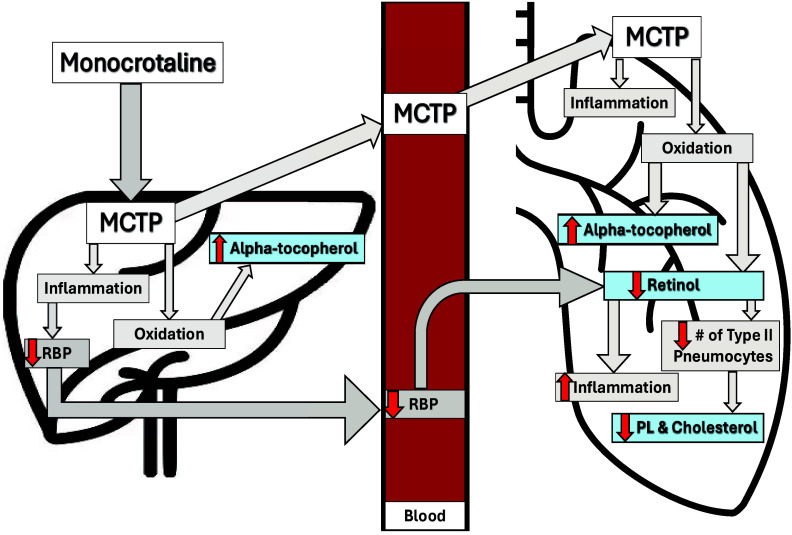
Schematic figure to summarize proposed mechanism of injury.

Our data supports a pathogenic model wherein MCT-induced oxidative and inflammatory insults within the lung trigger a localized depletion of retinol. Consequently, this micronutrient deficiency likely impairs the reparative and proliferative capacity of type II pneumocytes and may, in part, explain the observed reductions in total lung phospholipid and cholesterol (Figure 7). The systemic elevation of alpha-tocopherol appears to be a compensatory antioxidant response to this oxidative challenge. The hepatic retinol was unchanged, despite MCT’s known hepatotoxicity.

These findings suggest a novel mechanistic link between MCT-induced oxidative stress, lung vitamin A depletion, inflammation, and the impairment of alveolar cell proliferation and repair. This work identifies pulmonary retinol status as an important contributing factor in the pathogenesis of this model of lung injury.

## Supporting information

Baybutt et al. supplementary material Baybutt et al. supplementary material

## Supplementary material

The supplementary material for this article can be found at https://doi.org/10.1017/jns.2026.10077
